# Short Tandem Repeat Genotyping and Antifungal Susceptibility Testing of Latin American *Candida tropicalis* Isolates

**DOI:** 10.3390/jof9020207

**Published:** 2023-02-05

**Authors:** Bram Spruijtenburg, Cynthea C. S. Z. Baqueiro, Arnaldo L. Colombo, Eelco F. J. Meijer, João N. de Almeida, Indira Berrio, Norma B. Fernández, Guilherme M. Chaves, Jacques F. Meis, Theun de Groot, on behalf of the Latin American Group for Investigating Candida Tropicalis Resistance

**Affiliations:** 1 Department of Medical Microbiology and Infectious Diseases, Canisius-Wilhelmina Hospital, 6532 SZ Nijmegen, The Netherlands; 2 Center of Expertise in Mycology, Radboud University Medical Center, Canisius-Wilhelmina Hospital, 6532 SZ Nijmegen, The Netherlands; 3 Disciplina de Infectologia, Escola Paulista de Medicina, Universidade Federal de São Paulo, São Paulo 04021-001, SP, Brazil; 4 Hospital Israelita Albert Einstein, São Paulo 05652-900, SP, Brazil; 5 Hospital General de Medellín Luz Castro de Gutiérrez ESE, Medellín 050015, Colombia; 6 Medical and Experimental Mycology Group, Corporación para Investigaciones Biológicas (CIB), Medellín 050015, Colombia; 7 Hospital de Clínicas, Universidad de Buenos Aires, Buenos Aires 2351, Argentina; 8 Departamento de Análises Clínicas e Toxicológicas, Universidade Federal do Rio Grande do Norte, Natal 59078-970, RN, Brazil; 9 Bioprocess Engineering and Biotechnology Graduate Program, Federal University of Paraná, Curitiba 80060-000, PR, Brazil; 10 Department I of Internal Medicine, University of Cologne, Faculty of Medicine and University Hospital Cologne, Excellence Center for Medical Mycology, 50931 Cologne, Germany

**Keywords:** *Candida tropicalis*, genotyping, short tandem repeat, PCR, antifungal susceptibility

## Abstract

*Candida tropicalis* is emerging as one of the most common *Candida* species causing opportunistic infections in Latin America. Outbreak events caused by *C. tropicalis* were reported, and antifungal resistant isolates are on the rise. In order to investigate population genomics and look into antifungal resistance, we applied a short tandem repeat (STR) genotyping scheme and antifungal susceptibility testing (AFST) to 230 clinical and environmental *C. tropicalis* isolates from Latin American countries. STR genotyping identified 164 genotypes, including 11 clusters comprised of three to seven isolates, indicating outbreak events. AFST identified one isolate as anidulafungin-resistant and harboring a *FKS1* S659P substitution. Moreover, we identified 24 clinical and environmental isolates with intermediate susceptibility or resistance to one or more azoles. *ERG11* sequencing revealed each of these isolates harboring a Y132F and/or Y257H/N substitution. All of these isolates, except one, were clustered together in two groups of closely related STR genotypes, with each group harboring distinct *ERG11* substitutions. The ancestral *C. tropicalis* strain of these isolates likely acquired the azole resistance-associated substitutions and subsequently spread across vast distances within Brazil. Altogether, this STR genotyping scheme for *C. tropicalis* proved to be useful for identifying unrecognized outbreak events and better understanding population genomics, including the spread of antifungal-resistant isolates.

## 1. Introduction

*Candida tropicalis* is one of the most common causative species of candidemia, although epidemiology varies significantly in different parts of the world [1,2]. In parts of Latin American (LATAM) and Asian Pacific countries, *C. tropicalis* is the most common opportunistic *Candida* species, even surpassing *Candida albicans* and other non-*Candida albicans* candida (NCAC) species [3,4,5,6,7,8]. The yeast is considered the second most virulent after *C. albicans* and is reported as the most efficient biofilm producer compared to other pathogenic *Candida* species [9,10]. Besides *C. tropicalis* being part of the normal human microbiome, it is isolated from the environment, including from soil, plants, fresh water, marine ecosystems, and animals [7,11,12,13,14]. Globally, antifungal resistance is emerging, and *C. tropicalis* is no exception to this worrying trend [15,16,17,18,19]. Resistance to azoles, echinocandins, and amphotericin B were described [3,20,21,22,23]. Similar to other *Candida* species, upregulation of multidrug efflux pumps [22,24,25] and missense mutations in genes of the ergosterol pathway can lead to azole resistance, the latter primarily in the target *ERG11* [23,26,27,28,29]. In addition to azole resistance, azole trailing is occasionally observed and is defined as persistent but reduced growth above minimal inhibitory concentrations (MICs) [11,30,31]. Echinocandin resistance in *Candida* species is frequently caused by substitutions in the *FKS1* gene encoding 1,3-beta-D-glucan synthase, which is the target enzyme of echinocandins [32,33]. Resistance-associated substitutions are concentrated at so-called hot spot (HS) regions which echinocandins utilize as binding sites [15,34].

Most outbreaks caused by *C. tropicalis* were described in intensive care units (ICUs) [35,36]. Patients that are immunocompromised, receive broad-spectrum antibiotics, have indwelling catheters, are on hemodialysis, mechanical ventilation, and/or have had recent abdominal surgery are most notably at risk [10,37]. In order to investigate outbreaks and prevent further transmission, adequate genotyping is essential and could identify the source and transmission routes. Multiple genotyping schemes were developed for *C. tropicalis,* each with its own specific benefits and turn-offs. Multiple short tandem repeat (STR) genotyping schemes proved to have high resolution, short turnaround times, and high reproducibility. Moreover, STR genotyping assays were developed for *C. tropicalis* [38,39,40,41].

Previously, we developed STR genotyping for *C. tropicalis* and compared its outcomes with whole genome sequencing (WGS) single nucleotide polymorphism (SNP) analysis [42]. Here, we applied this STR genotyping scheme to a large collection of clinical and environmental Latin American (LATAM) isolates. Additionally, susceptibility testing and resistance-associated genes were investigated.

## 2. Materials and Methods

### 2.1. Isolates

All 230 *C. tropicalis* isolates, either clinical or environmental samples from LATAM countries, predominantly from Brazil, were collected between 2010 and 2022 (Table S1). Clinical isolates originated from blood cultures (taken from both peripheral blood and catheter), and environmental isolates were collected from beach sand (Ponta Negra Beach) in Natal, Brazil. Isolates were stored according to standard procedures at −80 °C. All isolates were identified by using matrix-assisted laser desorption ionization-time of flight (MALDI-TOF) mass spectrometry as previously described [43]. The ethics committee of the Federal University of São Paulo approved this study (Studies not enrolling human beings or animals; Approval code: 8260200319, 7 March 2019).

### 2.2. STR Genotyping and Data Analysis

All isolates were grown on Sabouraud dextrose agar (SDA) plates (Oxoid, Hampshire, United Kingdom) at 30 °C. A single colony was resuspended in 400 µL MagNA Pure bacteria lysis buffer and MagNA Lyser green beads (both Roche Diagnostics GmbH, Mannheim, Germany). The suspensions were mechanically lysed for 30 s at 6500 rpm using the MagNA Lyser (Roche Diagnostics). DNA was extracted and purified with the MagNA Pure 96 instrument and the MagNA Pure DNA and Viral NA Small Volume kit (Roche Diagnostics), following the manufacturer’s instructions and the Pathogen 200 SV protocol.

Multiplex PCR reactions amplifying six STR targets were performed on a thermocycler (Biometra, Westburg, Göttingen, Germany) using 1× FastStart *Taq* polymerase buffer without MgCl_2_, deoxynucleoside triphosphates (dNTPs) (0.2 mM), MgCl_2_ (3 mM), forward and reverse primers (1 to 5 µM), 1 U FastStart *Taq* polymerase (Roche Diagnostics), and isolated DNA. The thermal protocol for PCR amplification consisted of denaturation of 10 min at 95 °C followed by 30 cycles consisting of 30 sec denaturation at 95 °C, 30 s annealing at 60 °C, and 1 min of extension at 72 °C with a final incubation step for 10 min at 72 °C. PCR products were diluted 1:1000 in water, and 10 µL of the diluted product, in addition to 0.12 µL of the Orange 600 DNA size standard (NimaGen, Nijmegen, The Netherlands), was incubated for 1 min at 95 °C and analyzed on a 3500 XL genetic analyzer (Applied Biosystems, Foster City, CA, USA).

Corresponding copy numbers were determined using GeneMapper software (Applied Biosystems). Stutter peaks below 50% of the intensity of the highest peak for an allele, minus-A peaks and bleed-through peaks, were discarded for all markers. Copy numbers were ensured to be rounded. Copy numbers were converted to a binary matrix: “1” if an isolate contained the allele and “0” if it did not. The relatedness between isolates was analyzed with BioNumerics v.7.6.1 software (Applied Maths NV, Sint-Martens-Latem, Belgium), utilizing the unweighted pair group method with arithmetic mean averages (UPGMA) and using the multistate categorical similarity coefficient as previously described [44].

### 2.3. Antifungal Susceptibility Testing (AFST) and Resistance-Associated Gene Investigation

In vitro AFSTs against fluconazole (MERK, Darmstadt, Germany), voriconazole (MERK, Darmstadt, Germany), amphotericin B (MERK, Darmstadt, Germany), and anidulafungin (MERK, Darmstadt, Germany) were performed via broth microdilution according to the Clinical and Laboratory Standard Institute (CLSI) M27-S4 (23). In short, colonies were diluted in RPMI medium, and final concentrations of 1 × 10^3^–5 × 10^5^ CFU/mL were obtained with a Genesys 20 Spectrophotometer (Thermo Fisher Scientific, Waltham, MA, USA). Microtiter plates were incubated at 35 °C and visually interpreted after 24 h. MIC values were read as the lowest antifungal concentration with a 50% growth reduction when compared to the growth control, except for amphotericin B with a 100% growth reduction. Interpretive categories were implemented according to the definition by CLSI [45]. For fluconazole, MIC breakpoints of ≤2, 4, and ≥8 mg/L were considered susceptible, susceptible-dose dependent, and resistant, respectively. For voriconazole, ≤0.125, 0.25–0.5, and ≥1 mg/L were indicated as susceptible, intermediate, and resistant, respectively. Anidulafungin breakpoints were ≤0.25, 0.5, and ≥1 mg/L for susceptible, intermediate, and resistant, respectively. Amphotericin B breakpoints were set as ≤1 mg/L for susceptible and ≥1 mg/L as resistant. Trailing was optically interpreted as persistent but with residual growth above the MIC value compared to the growth control [30,31]. *ERG11* and FKS1 gene sequences were amplified as previously described [25,33]. Amplified DNA was purified using the D-pure purification protocol (Nimagen), sequenced on a 3500 XL genetic analyzer (Applied Biosystems), and analyzed using BioNumerics 7.6.1 (Applied Maths). Generated sequences of susceptible and resistant isolates were compared to each other with the use of the multiple alignment option with an open gap penalty of 100%. Lanosterol 14 α-demethylase (*ERG11*) protein homology modelling was conducted with SWISS-MODEL (https://swissmodel.expasy.org/interactive, accessed on 2 November 2022), by using the 5eqb.1A “Crystal structure of lanosterol 14 α demethylase (*ERG11*) with intact transmembrane domain bound to itraconazole” template [46]. The predicted lanosterol 14 α-demethylase (*ERG11*) protein model was visually inspected to ensure that all major P450 structural motifs (I-helix, FG loop, and the substrate access channel) were intact. The resulting PDB file was uploaded to PyMOL v2.0, and the amino acids Y132 and Y257 were subsequently marked red [47].

## 3. Results

### 3.1. Application of C. tropicalis STR Genotyping

**Figure 1:**
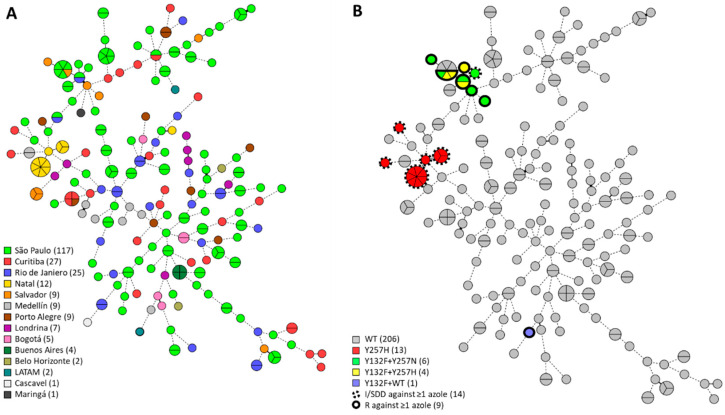
**Minimum-spanning tree of 230 *C. tropicalis* isolates.** Branch lengths indicate the similarity between isolates with thick solid lines (variation in one allele), thin solid lines (variation in two alleles), and thin dotted lines (variation in three or more alleles). Azole-intermediate and azole-resistant isolates are demarcated in dotted black lines and solid black lines, respectively. On the left (**A**), the number of isolates per city are shown in the color key; on the right (**B**), isolates with *ERG11* substitutions and reduced azole susceptibility are shown as in the color key. LATAM, Latin American.

A STR genotyping for *C. tropicalis* was applied to all 230 *C. tropicalis* isolates as previously identified by MALDI-TOF. Isolates were predominantly from São Paulo (n = 117, 50.9%), Curitiba (n = 27, 11.7%), and Rio de Janeiro (n = 25, 10.9%). Genotyping resulted in the identification of 164 different genotypes each consisting of one to seven isolates (Figure 1A). Overall genetic diversity was high, with the vast majority of isolates exhibiting copy number differences in more than three microsatellite markers. We found 32 small clusters of two isolates in addition to 11 large clusters with more than two isolates. Isolates from large clusters exclusively originated from São Paulo, Natal, Curitiba, Salvador, and Buenos Aires. Five of these large clusters spanned two different cities; 18 of 32 small clusters comprised two independent isolates taken from the same patient. All but one of the environmental isolates (ID: 1443/2017) from Ponta Negra Beach, Natal, Brazil (n = 12, 5.2%) exhibited highly similar genotypes (Gt 95 to 97) and were closely related to clinical isolates (Gt 93 and 94) with copy number differences in three alleles (Figure S1).

### 3.2. AFST and Resistance-Associated Genes Investigation

AFST CLSI microbroth dilution was performed on all isolates using fluconazole, voriconazole, amphotericin B, and anidulafungin (Table S1). Elevated MICs against azoles and anidulafungin were found in 10.4% of all isolates, those being intermediate (6.1%; n = 15) or resistant (3.9%; n = 9) to at least one azole drug, and one isolate (0.4%) was found to be resistant to anidulafungin (Table 1). Trailing was observed in two isolates (IDs: 7225/2010 and 1535/2017) with reduced susceptibility to azoles (Table 2). *ERG11* and *FKS1* genes were sequenced to identify mutations conferring resistance. Y132F, Y257H, and Y257N substitutions in *ERG11* were found exclusively in isolates with reduced susceptibility to at least one azole drug (Table 2). The azole-intermediate and -resistant isolates all originated from Brazil, except for one Colombian isolate (ID: 291/2018). These resistant Brazilian isolates were confined to two groups of related STR genotypes, despite isolates from both groups originating from diverse cities up to 2,600 km apart. One of these groups (n = 13, IDs: 7225/2010, 7416/2011, 1450/2017, 1452/2017, 1453/2017, 1463/2017, 1468/2017, 1471/2017, 1483/2017, 1486/2017, 1488/2017, 1491/2017, and 1498/2017) was solely intermediately resistant to azoles, harbored the *ERG11* Y257H substitution, and included environmental isolates (n = 11). The other group comprised intermediate and resistant isolates (n = 10, IDs: 8095/2011, 9253/2013, 822/2015, 1534/2017, 1535/2017, 1743/2017, 681/2019, 683/2019, 1540/2020, and 1541/2020) and exhibited the *ERG11* Y132F and Y257H/Y257N substitutions. The fluconazole-resistant Colombian isolate was not closely related to these two groups of intermediate and resistant isolates, and it harbored a heterozygous *ERG11* Y132F substitution, whereas all previously mentioned substitutions were homozygous (Figure 1B, Table 2). Protein homology modelling for lanosterol 14 α-demethylase (*ERG11*) was conducted to visualize and inspect the resistance-associated substitutions (Figure S2). The *ERG11* Y257H/N substitution was situated in the G-helix, which is at the rear of the protein at the putative substrate access channel. The echinocandin resistant isolate (ID: 1751/2017) exhibited a *FKS1* S659P substitution located in HS-1 (Table 2).

## 4. Discussion

In this study, an STR genotyping scheme for *C. tropicalis* was applied to 230 clinical and environmental *C. tropicalis* isolates from Latin American countries. STR genotyping identified 164 different genotypes, indicating an overall high genetic diversity within LATAM countries, especially in Brazil. Limited data is available regarding *C. tropicalis* phylogenetic relationships within LATAM countries. One Brazilian study was in line with the present observation of an overall high genetic diversity, according to MLST genotyping [48]. In total, 11 large clusters consisting of more than two isolates were found. Using AFSTs of fluconazole, voriconazole, amphotericin B, and anidulafungin, 15 isolates were found to have intermediately resistant MICs to at least one azole drug, and nine isolates were resistant to at least one azole drug. Except for one isolate with a reduced susceptibility to azoles, all were confined to two groups of related STR genotypes. These isolates all harbored one or more of the azole resistance-associated substitutions *ERG11* Y132F, Y257H, and Y257N. Additionally, one isolate was resistant to anidulafungin and exhibited an *FKS1* S659P substitution in HS1.

### 4.1. Application of C. tropicalis STR Genotyping

Within the 164 different *C. tropicalis* genotypes, we found several clusters of varying sizes comprising two to seven isolates from different patients, suggesting outbreak events within multiple hospitals. The majority of these previously unrecognized outbreak events included isolates collected within a few months from each other, suggesting the possibility of persistent clonal spread, as was also found for *C. parapsilosis* in Brazilian hospital settings [8,49,50]. Multiple studies have identified *C. tropicalis* as the strongest biofilm producer when compared to other pathogenic *Candida* species, making this yeast highly capable of colonizing intravascular lines and devices [9,10,51,52]. Moreover, multiple studies identified healthcare personnel as the source of *C. tropicalis* outbreaks [35,53,54]. From 38 patients, two samples were taken on consecutive dates, and genotypes were identical. For nine patients, one sample was cultured from peripheral blood and one from catheter blood, both samples always exhibiting the same STR genotype. Taken together with the short timespan between these related infections within the same hospital wards, it is possible that nosocomial spread due to inadequate infection prevention and control measures was involved in these outbreaks.

Interestingly, five clusters represented isolates from two different cities up to 1.400 km apart from each other. These findings suggest the spread of multiple *C. tropicalis* genotypes across a vast distance, but WGS data analysis is necessary to confirm that hypothesis. The environmental isolates from Natal, Brazil were recovered from sand at the Ponta Negra beach, which is a popular recreational destination. Previously, *C. tropicalis* isolates were isolated from the same beach, and some were found to be resistant to azoles [13]. The environmental isolates in this study were related to clinical samples from Brazil and Colombia, suggesting the dissemination of *C. tropicalis* isolates due to human activities, as was previously also shown for *C. tropicalis* and *C. auris* [55,56]. Our findings highlight the importance of high-resolution genotyping to reveal outbreak events within hospitals and obtain a better understanding of population genomics.

### 4.2. AFST and Resistance Associated Genes Investigation

AFST identified 24 isolates (10.4%) that were intermediate or resistant to one or more azole drugs, in addition to one isolate (0.4%) being resistant to anidulafungin. Interestingly, all Brazilian isolates with a reduced susceptibility to azoles were confined to two separate groups of closely related STR genotypes despite originating from cities far apart. All isolates from the first group harbored the *ERG11* Y257H substitution and were intermediately resistant to at least one azole drug. The absence of this mutation in other isolates suggests that spontaneous development of this mutation is unlikely and that this mutation may have originated from a common single ancestor *C. tropicalis* strain in Brazil. Widespread use of agricultural azoles is known to have driven the emergence of environmental resistance mechanisms in fungi, which could hypothetically be a contributing factor in this setting [57,58,59,60,61]. However, although 11 out of 23 of these isolates originated from the environment, a non-environmental source cannot be excluded, as these samples were acquired from a public beach. The *ERG11* Y257H substitution was previously associated with reduced fluconazole susceptibility in *C. tropicalis* and *C. albicans* [62,63]. The MICs found in this study indicate the Y257H substitution lowers the susceptibility to azoles to an intermediate level. In *C. albicans*, the Y257H was thought to prevent fluconazole from entering the active site, leading to reduced susceptibility. The *ERG11* Y257 in *C. tropicalis* and *C. albicans* correspond to the same position and are located in the G-helix; therefore, the mode of action in *C. albicans* is likely identical for *C. tropicalis* [28,42,64,65].

Resistant isolates in the other group of related STR genotypes all harbored an *ERG11* Y132F substitution in combination with a Y257H/N substitution. Again, the unique presence of the Y132F mutation of this group as compared to the other *C. tropicalis* isolates in this study suggests that the mutation originated from an ancestor strain of this group, whereas the Y257H or Y257N substitutions were likely acquired more recently. The Y257H/N substitution might prevent fluconazole from entering the binding site because aromatic rings in asparagine and histidine are absent there but present in tyrosine. Aromatic rings of amino acids such as tyrosine form pi–pi binding interactions with other aromatic rings, as also present in fluconazole [66]. This potential interaction between the aromatic rings is postulated to facilitate the binding between azoles and *ERG11,* leading to reduced protein activity and, therewith, increased azole susceptibility [65,67]. Finally, one STR cluster of six isolates within this group contained three resistant and three susceptible isolates. In the latter three, substitutions conferring azole resistance were absent. These susceptible isolates all originated from one patient and were collected in 2021, whereas the resistant isolates were collected in 2019 and 2020. These three recent isolates might have lost the Y132F and Y257H/N substitutions in a patient or an environment without drug pressure, as azole resistance is also associated with a loss of fitness [16,68].

The present study has some limitations. Previously, STR and WGS SNP outcomes were highly concordant, indicating a high resolution for the STR typing scheme, but the exact resolution is unknown and might differ in the present isolate collection. Moreover, only the *ERG11* gene was sequenced, without taking into account other resistance-associated genes like *UPC2* and *TAC1B*, which could harbor mutations conferring resistance. In a follow-up study, WGS could be used to identify more mutations in resistance-associated genes and confirm the close relatedness of these isolates according to STR typing despite originating from diverse cities. Additionally, extensive environmental sampling and genotyping could trace the source of the clusters found in this study. The limited clinical data in the present study hampered the interpretation of genotypic clustering.

To summarize, we applied an STR genotyping scheme for *C. tropicalis* to clinical and environmental isolates from LATAM countries to identify clusters of previously unrecognized outbreak events. AFST identified 10% of isolates with reduced susceptibility to azoles. Except for one isolate, all were confined to two related groups of STR genotypes, and all isolates harbored resistance-associated substitutions in *ERG11*. Each group exhibited a different set of substitutions, suggesting an ancestral *C. tropicalis* strain in both groups that subsequently spread to diverse cities across vast distances. Only one isolate was anidulafungin-resistant and exhibited a *FKS1* S659P substitution in HS1, reflecting the low prevalence of *C. tropicalis* echinocandin-resistance in LATAM countries.

## Figures and Tables

**Table 1:** **MIC ranges, GM, MIC_50_, and MIC_90_ values found performing microbroth dilution for 24 h according to the CLSI M27-S4 standard.** MIC, minimal inhibitory concentration; GM, geometric mean.

Antifungal	Range (mg/L)	GM (mg/L)	MIC_50_ (mg/L)	MIC_90_ (mg/L)
Fluconazole	0.125–8	0.83	0.25	4
Voriconazole	0.03–1	0.08	0.03	0.25
Amphotericin B	0.125–1	0.56	0.5	1
Anidulafungin	0.03–1	0.04	0.03	0.06

**Table 2:** **Overview of isolates resistant or intermediately resistant to azoles or echinocandins with corresponding *ERG11* or FKS1 substitutions.** MIC, minimal inhibitory concentration; FLC, fluconazole; VOR, voriconazole; AFG, anidulafungin.

ID	FLC MIC	VOR MIC	AFG MIC	*ERG11* Substitution	*FKS1* Substitution
7225/2010	4 *	0.25 *	0.03	Y257H	-
7416/2011	4	0.25	0.125	Y257H	-
8095/2011	4	0.125	0.06	Y132F, Y257H	-
9253/2013	8	0.5	0.06	Y132F, Y257N	-
822/2015	8	1	0.06	Y132F, Y257N	-
1450/2017	4	0.25	0.06	Y257H	-
1452/2017	4	0.25	0.06	Y257H	-
1453/2017	4	0.25	0.06	Y257H	-
1463/2017	4	0.25	0.06	Y257H	-
1468/2017	4	0.25	0.06	Y257H	-
1471/2017	4	0.25	0.06	Y257H	-
1483/2017	4	0.25	0.06	Y257H	-
1486/2017	4	0.25	0.06	Y257H	-
1488/2017	2	0.25	0.06	Y257H	-
1491/2017	4	0.25	0.06	Y257H	-
1498/2017	2	0.25	0.06	Y257H	-
1534/2017	8	0.5	0.03	Y132F, Y257N	-
1535/2017	8 *	0.5 *	0.03	Y132F, Y257N	-
1743/2017	4	0.5	0.03	Y132F, Y257H	-
1751/2017	0.5	0.06	1	-	S659P
291/2018	8	0.25	0.03	Y132F ^†^	-
681/2019	8	0.5	0.03	Y132F, Y257H	-
683/2019	8	1	0.03	Y132F, Y257H	-
1540/2020	8	0.5	0.03	Y132F, Y257N	-
1541/2020	8	0.5	0.06	Y132F, Y257N	-

* Trailing was observed when MICs were visually interpreted; ^†^ Y132F substitution is heterozygous, with one allele being WT and the other being Y132F.

## Data Availability

Data sharing is not applicable.

## Supplementary Materials

The following supporting information can be downloaded at: https://www.mdpi.com/article/10.3390/jof9020207/s1, Figure S1: Cluster analysis of 230 *C. tropicalis* isolates. Branch lengths indicate relatedness according to microsatellite alleles. The UPGMA dendrogram was generated with BioNumerics 7.6.1 (Applied Maths, Kortrijk, Belgium) using the multistate categorical similarity coefficient. Bootstrap values using 1000 replicates were above 95% or specified at the branch when lower. Gt, genotype; FLC, fluconazole; S, susceptible; SDD, susceptible-dose dependent; R, resistant; Figure S2: *C. tropicalis* lanosterol 14 α-demethylase (*ERG11*) homology model. This model was constructed with SWISS-MODEL and viewed from multiple angles. Front view (A), side view (B), and top view (C), with the Y132 and Y257 amino acids marked in red. Y132 is located near the core of the protein, whereas Y257 is located in the G-helix at the rear of the protein. Table S1: Overview of all *C. tropicalis* isolates, including AFST MICs according to microbroth dilutions performed to the CLSI M27-S4 standard and clinical details. Gt, genotype; FLC, fluconazole; VRC, voriconazole; AMB, amphotericin B; AFG, anidulafungin; ICU, intensive care unit.
