# Supplementary material for: Short Tandem Repeat Genotyping and Antifungal Susceptibility Testing of Latin American Candida tropicalis Isolates
Source: J Fungi (Basel). 2023 Feb 5;9(2):207. doi: 10.3390/jof9020207 (PMC9958743; doi:10.3390/jof9020207)
Supplement: Supplementary file 1 [file jof-09-00207-s001.zip › jof-2189999-supplementary/Table S1.pdf]

**Table S1: Overview of all *C. tropicalis* isolates, including AFST MICs according to microbroth dilutions CLSI M27-S4 standard and clinical details.** Gt, genotype; FLC, fluconazole; VRC, voriconazole; AMB, amphotericin B; AFG, anidulafungin; ICU, intensive care unit

| ID | Country | City | Hospital (#) | Hospital unit | Collection site* | Year of isolation | Gt | FLC | VRC | AMB | AFG |
|---|---|---|---|---|---|---|---|---|---|---|---|
| 1150/2016 | Brazil | Londrina | 1 | Unknown | Blood culture | 2016 | 1 | 1 | 0.03 | 0.5 | 0.03 |
| 1153/2016 | Brazil | Londrina | 1 | Unknown | Blood culture | 2016 | 2 | 2 | 0.03 | 0.5 | 0.03 |
| CMPR5544 | Brazil | Curitiba | 2 | Unknown | Blood culture | 2022 | 3 | 1 | 0.03 | 0.5 | 0.06 |
| 1557/2020 | Brazil | Salvador | 3 | Pediatric ICU | Blood culture | 2019 | 4 | 0.25 | 0.03 | 0.25 | 0.03 |
| 1559/2020 | Brazil | Salvador | 3 | Pediatric ICU | Blood culture | 2019 | 4 | 0.25 | 0.03 | 0.5 | 0.03 |
| 1681/2020 | Brazil | Salvador | 3 | ICU II immune-compromised | Blood culture | 2020 | 4 | 1 | 0.03 | 0.5 | 0.03 |
| 8295/2011 | Brazil | Porto Alegre | 4 | Surgery | Blood culture | 2011 | 5 | 0.5 | 0.06 | 0.25 | 0.03 |
| 7394A/2011 | Brazil | Belo Horizonte | 5 | General ICU | Blood culture | 2011 | 6 | 0.125 | 0.03 | 0.25 | 0.03 |
| 1189/2020 | Brazil | São Paulo | 6 | Unknown | Blood culture | 2020 | 7 | 0.125 | 0.03 | 0.5 | 0.03 |
| 1248A/2016 | Brazil | Londrina | 7 | Unknown | Blood culture | 2016 | 8 | 0.125 | 0.03 | 0.5 | 0.03 |
| 8083/2011 | Brazil | Curitiba | 2 | Emergency | Blood culture | 2011 | 9 | 0.25 | 0.03 | 0.25 | 0.03 |
| 8088/2011 | Brazil | Curitiba | 2 | Surgery | Blood culture | 2011 | 10 | 0.25 | 0.03 | 0.25 | 0.03 |
| 7052/2010 | Colombia | Bogotá | 8 | Unknown | Blood culture | 2010 | 11 | 0.25 | 0.03 | 0.05 | 0.06 |
| 7053/2010 | Colombia | Bogotá | 8 | Unknown | Blood culture | 2010 | 12 | 0.25 | 0.03 | 1 | 0.06 |
| 291/2018 | Colombia | Medellín | 9 | Unknown | Blood culture | 2018 | 13 | 8 | 0.25 | 0.5 | 0.03 |
| 6988/2010 | LATAM | Unknown | 10 | Unknown | Blood culture | 2010 | 14 | 0.125 | 0.03 | 1 | 0.03 |
| CMRP5546 | Brazil | Curitiba | 2 | Unknown | Blood culture | 2022 | 15 | 0.25 | 0.03 | 0.5 | 0.06 |
| CMRP5548 | Brazil | Curitiba | 2 | Unknown | Blood culture | 2022 | 16 | 0.125 | 0.03 | 1 | 0.03 |
| CMRP5552 | Brazil | Curitiba | 2 | Unknown | Blood culture | 2022 | 17 | 0.5 | 0.06 | 0.5 | 0.06 |
| CMRP5542 | Brazil | Curitiba | 2 | Unknown | Blood culture | 2022 | 18 | 0.25 | 0.03 | 0.25 | 0.03 |
| 8078/2011 | Brazil | São Paulo | 11 | Surgical ICU | Blood culture | 2011 | 19 | 0.125 | 0.03 | 0.5 | 0.03 |
| 1737/2017 | Brazil | Rio de Janeiro | 12 | Unknown | Blood culture | 2017 | 20 | 0.125 | 0.03 | 0.25 | 0.03 |
| 7083/2010 | Brazil | Curitiba | 2 | Unknown | Blood culture | 2010 | 21 | 0.25 | 0.03 | 0.5 | 0.03 |
| 7232B/2010 | Brazil | Curitiba | 2 | Unknown | Blood culture | 2010 | 21 | 0.25 | 0.06 | 1 | 0.06 |
| 7459A/2011 | Brazil | Salvador | 3 | Coronary Unit ICU | Blood culture | 2011 | 22 | 0.125 | 0.03 | 0.5 | 0.03 |
| 167/2019 | Brazil | São Paulo | 3 | Medical clinical ICU | Blood culture | 2019 | 23 | 0.25 | 0.03 | 0.25 | 0.03 |
| 168/2019 | Brazil | São Paulo | 3 | Medical clinical ICU | Blood culture | 2019 | 23 | 0.25 | 0.03 | 0.5 | 0.03 |
| 1877/2021 | Brazil | São Paulo | 6 | Unknown | Blood culture | 2021 | 23 | 0.125 | 0.03 | 0.5 | 0.03 |
| 1741A0/2017 | Brazil | Rio de Janeiro | 12 | Unknown | Blood culture | 2013 | 24 | 0.125 | 0.03 | 0.25 | 0.03 |
| 1741B0/2017 | Brazil | Rio de Janeiro | 12 | Unknown | Blood culture | 2013 | 24 | 0.125 | 0.03 | 0.5 | 0.03 |
| 510/2019 | Brazil | São Paulo | 13 | Unknown | Blood culture | 2018 | 25 | 0.125 | 0.03 | 0.25 | 0.03 |
| 1734B0/2017 | Brazil | Rio de Janeiro | 12 | Unknown | Blood culture | 2011 | 26 | 0.125 | 0.03 | 0.5 | 0.03 |
| 1735C0/2017 | Brazil | Rio de Janeiro | 12 | Unknown | Blood culture | 2011 | 26 | 0.125 | 0.03 | 0.5 | 0.03 |
| 483/2019 | Brazil | Curitiba | 2 | Surgical nursery | Blood culture | 2018 | 27 | 0.125 | 0.03 | 0.25 | 0.03 |
| 284/2015 | Brazil | São Paulo | 3 | ICU UPOCC | Catheter blood | 2015 | 28 | 0.125 | 0.03 | 0.5 | 0.03 |
| 301/2015 | Brazil | São Paulo | 3 | ICU burn unit | Blood culture | 2015 | 28 | 0.125 | 0.03 | 0.5 | 0.03 |
| 1381/2020 | Brazil | São Paulo | 3 | General ICU Adult II | Blood culture | 2020 | 29 | 0.125 | 0.03 | 0.5 | 0.03 |
| 305/2015 | Brazil | São Paulo | 6 | Unknown | Blood culture | 2015 | 30 | 0.125 | 0.03 | 0.5 | 0.03 |
| 1443/2017 | Brazil | Natal | Non applicable | Non applicable | Ponta Negra beach | 2017 | 31 | 1 | 0.06 | 0.5 | 0.03 |
| 6685/2010 | Brazil | Rio de Janeiro | 15 | Unknown | Blood culture | 2010 | 32 | 0.25 | 0.03 | 0.5 | 0.03 |
| 7048/2010 | Colombia | Bogotá | 16 | Unknown | Blood culture | 2010 | 33 | 0.5 | 0.06 | 0.25 | 0.03 |
| 486/2018 | Brazil | São Paulo | 3 | ICU burn unit | Blood culture | 2018 | 34 | 0.125 | 0.03 | 0.5 | 0.03 |
| 8080A/2011 | Brazil | São Paulo | 11 | Pediatric ICU | Blood culture | 2011 | 35 | 0.125 | 0.03 | 0.5 | 0.03 |
| 8080C/2011 | Brazil | São Paulo | 11 | Pediatric ICU | Blood culture | 2011 | 35 | 0.25 | 0.03 | 0.125 | 0.03 |
| 780/2019 | Brazil | Rio de Janeiro | 15 | Unknown | Blood culture | 2018 | 36 | 0.125 | 0.03 | 0.5 | 0.03 |
| 8142/2011 | Argentina | Buenos Aires | 17 | Unknown | Blood culture | 2010 | 37 | 0.125 | 0.03 | 0.5 | 0.03 |
| 8146A/2011 | Argentina | Buenos Aires | 17 | Unknown | Blood culture | 2010 | 37 | 0.125 | 0.03 | 0.5 | 0.03 |

| | | | | | | | | | | | |
|---|---|---|---|---|---|---|---|---|---|---|---|
| 8146B/2011 | Argentina | Buenos Aires | 17 | Unknown | Blood culture | 2010 | 37 | 0.125 | 0.03 | 0.5 | 0.06 |
| 8141/2011 | Argentina | Buenos Aires | 17 | Unknown | Blood culture | 2010 | 37 | 0.125 | 0.03 | 0.25 | 0.03 |
| 1178/2020 | Brazil | São Paulo | 18 | Unknown | Blood culture | 2020 | 38 | 0.125 | 0.03 | 0.5 | 0.03 |
| 1179/2020 | Brazil | São Paulo | 18 | Unknown | Catheter blood | 2020 | 38 | 0.25 | 0.03 | 0.5 | 0.03 |
| 491/2018 | Brazil | São Paulo | 6 | Unknown | Blood culture | 2018 | 39 | 0.125 | 0.03 | 0.5 | 0.03 |
| 1883/2021 | Brazil | São Paulo | 19 | Orthopedics ICU | Blood culture | 2021 | 40 | 0.125 | 0.03 | 0.5 | 0.03 |
| 8893/2012 | Brazil | São Paulo | 6 | Unknown | Blood culture | 2012 | 41 | 0.25 | 0.03 | 0.5 | 0.06 |
| 8921E/2012 | Brazil | São Paulo | 6 | Unknown | Blood culture | 2012 | 41 | 0.25 | 0.03 | 0.125 | 0.03 |
| 10105/2014 | Brazil | São Paulo | 6 | Unknown | Blood culture | 2014 | 42 | 0.125 | 0.03 | 0.5 | 0.06 |
| 630/2019 | Brazil | São Paulo | 19 | General ICU | Blood culture | 2017 | 43 | 0.125 | 0.03 | 0.5 | 0.03 |
| 6336A/2010 | Brazil | São Paulo | 3 | Medical clinical ICU | Blood culture | 2010 | 44 | 0.125 | 0.03 | 1 | 0.03 |
| 6336B/2010 | Brazil | São Paulo | 3 | Medical clinical ICU | Catheter blood | 2010 | 44 | 0.25 | 0.03 | 1 | 0.06 |
| 7809/2011 | Brazil | São Paulo | 11 | Pediatric ICU | Blood culture | 2011 | 45 | 0.25 | 0.03 | 0.5 | 0.03 |
| 722/2019 | Brazil | Porto Alegre | 4 | Surgical nursery | Blood culture | 2018 | 46 | 0.125 | 0.03 | 0.25 | 0.03 |
| 298/2018 | Colombia | Medellín | 9 | Unknown | Blood culture | 2018 | 47 | 0.25 | 0.06 | 0.5 | 0.06 |
| 228/2015 | Brazil | São Paulo | 6 | Unknown | Blood culture | 2015 | 48 | 1 | 0.03 | 0.5 | 0.03 |
| 1753/2017 | Brazil | Rio de Janeiro | 12 | Unknown | Blood culture | 2015 | 49 | 0.125 | 0.03 | 0.5 | 0.03 |
| 297/2018 | Colombia | Medellín | 9 | Unknown | Blood culture | 2018 | 50 | 2 | 0.125 | 1 | 0.03 |
| 299/2018 | Colombia | Medellín | 9 | Unknown | Blood culture | 2018 | 51 | 1 | 0.125 | 1 | 0.03 |
| 9262/2013 | Brazil | São Paulo | 3 | ICU adult first aid | Blood culture | 2013 | 52 | 0.5 | 0.06 | 1 | 0.06 |
| 9274A/2013 | Brazil | São Paulo | 3 | General ICU adult | Blood culture | 2013 | 52 | 0.5 | 0.06 | 1 | 0.06 |
| 9274B/2013 | Brazil | São Paulo | 3 | General ICU adult | Catheter blood | 2013 | 52 | 0.5 | 0.06 | 1 | 0.06 |
| 9275B/2013 | Brazil | São Paulo | 6 | Unknown | Blood culture | 2013 | 53 | 0.25 | 0.03 | 0.5 | 0.03 |
| 9275C/2013 | Brazil | São Paulo | 6 | Unknown | Blood culture | 2013 | 53 | 0.5 | 0.03 | 0.5 | 0.03 |
| 1761/2017 | Brazil | Rio de Janeiro | 12 | Unknown | Blood culture | 2016 | 54 | 0.25 | 0.03 | 1 | 0.03 |
| 745/2019 | Brazil | Porto Alegre | 4 | Unknown | Blood culture | 2017 | 55 | 0.125 | 0.03 | 0.5 | 0.03 |
| 8068/2011 | Brazil | Curitiba | 20 | Emergency | Blood culture | 2010 | 56 | 0.125 | 0.03 | 0.5 | 0.03 |
| 1110/2016 | Brazil | São Paulo | 18 | Unknown | Catheter blood | 2016 | 57 | 0.25 | 0.03 | 0.5 | 0.03 |
| 7550A/2011 | Brazil | Curitiba | 21 | Medical clinic | Blood culture | 2010 | 58 | 0.125 | 0.03 | 0.5 | 0.03 |
| 339/2019 | Brazil | São Paulo | 6 | Unknown | Blood culture | 2019 | 59 | 0.125 | 0.03 | 0.5 | 0.03 |
| 963/2019 | Brazil | São Paulo | 3 | ICU adult first aid | Catheter blood | 2019 | 59 | 0.125 | 0.03 | 0.5 | 0.03 |
| 7013/2010 | Colombia | Bogotá | 22 | Unknown | Blood culture | 2010 | 60 | 0.125 | 0.03 | 1 | 0.03 |
| 7043/2010 | Colombia | Bogotá | 22 | Unknown | Blood culture | 2010 | 60 | 0.125 | 0.03 | 1 | 0.03 |
| 1754/2017 | Brazil | Rio de Janeiro | 12 | Unknown | Blood culture | 2015 | 61 | 0.25 | 0.03 | 0.5 | 0.03 |
| 2240/2021 | Brazil | São Paulo | 3 | General ICU adult II | Blood culture | 2021 | 62 | 0.125 | 0.03 | 0.5 | 0.03 |
| 2241/2021 | Brazil | São Paulo | 3 | General ICU adult II | Blood culture | 2021 | 62 | 0.125 | 0.03 | 0.25 | 0.03 |
| CMRP5554 | Brazil | Curitiba | 2 | Unknown | Blood culture | 2022 | 63 | 0.125 | 0.03 | 0.5 | 0.03 |
| CMRP5551 | Brazil | Curitiba | 2 | Unknown | Blood culture | 2022 | 64 | 0.25 | 0.03 | 0.25 | 0.03 |
| 2017/2021 | Brazil | São Paulo | 3 | Observation unit adult first aid | Blood culture | 2021 | 65 | 0.5 | 0.03 | 0.25 | 0.03 |
| 310/2018 | Brazil | São Paulo | 18 | Unknown | Catheter blood | 2018 | 66 | 0.125 | 0.03 | 0.5 | 0.03 |
| 335/2018 | Brazil | São Paulo | 18 | Unknown | Catheter blood | 2018 | 66 | 0.125 | 0.03 | 0.5 | 0.03 |
| 91/2019 | Brazil | São Paulo | 18 | Unknown | Blood culture | 2019 | 67 | 0.25 | 0.03 | 0.5 | 0.06 |
| 92/2019 | Brazil | São Paulo | 8 | Unknown | Blood culture | 2019 | 67 | 0.25 | 0.03 | 0.5 | 0.03 |
| 139/2015 | Brazil | São Paulo | 3 | IU gastrosurgery | Blood culture | 2015 | 68 | 2 | 0.125 | 1 | 0.06 |
| 141/2015 | Brazil | São Paulo | 3 | IU gastrosurgery | Blood culture | 2015 | 68 | 0.5 | 0.06 | 0.5 | 0.06 |
| 178/2015 | Brazil | São Paulo | 3 | IU gastrosurgery | Blood culture | 2015 | 68 | 0.5 | 0.06 | 1 | 0.06 |
| 340/2019 | Brazil | São Paulo | 18 | Unknown | Blood culture | 2019 | 69 | 0.25 | 0.03 | 0.5 | 0.03 |
| 360/2019 | Brazil | São Paulo | 18 | Unknown | Blood culture | 2019 | 69 | 0.25 | 0.03 | 0.5 | 0.03 |
| 1752B0/2017 | Brazil | Rio de Janeiro | 12 | Unknown | Blood culture | 2017 | 70 | 0.25 | 0.03 | 0.5 | 0.03 |
| 1752C0/2017 | Brazil | Rio de Janeiro | 12 | Unknown | Blood culture | 2017 | 70 | 0.25 | 0.03 | 0.5 | 0.03 |
| 2196/2021 | Brazil | São Paulo | 3 | General ICU adult II | Blood culture | 2021 | 71 | 0.5 | 0.03 | 1 | 0.03 |

| | | | | | | | | | | | |
|---|---|---|---|---|---|---|---|---|---|---|---|
| 1922/2017 | Brazil | São Paulo | 3 | Medical clinical ICU | Blood culture | 2017 | 72 | 0.25 | 0.03 | 0.5 | 0.03 |
| 7244A/2010 | Brazil | Curitiba | 2 | Unknown | Blood culture | 2010 | 73 | 0.125 | 0.03 | 0.5 | 0.03 |
| 7244C/2010 | Brazil | Curitiba | 2 | Unknown | Blood culture | 2010 | 73 | 0.25 | 0.03 | 1 | 0.06 |
| 7258A/2010 | Brazil | Curitiba | 2 | Pediatric infection | Blood culture | 2010 | 73 | 0.25 | 0.03 | 0.5 | 0.03 |
| 7619/2011 | Brazil | Porto Alegre | 4 | Medical clinic | Blood culture | 2010 | 73 | 0.5 | 0.06 | 0.5 | 0.03 |
| 241/2019 | Brazil | São Paulo | 3 | ICU pediatrics | Blood culture | 2019 | 74 | 0.125 | 0.03 | 0.5 | 0.03 |
| 1135/2016 | Brazil | Curitiba | 23 | Unknown | Blood culture | 2016 | 75 | 0.125 | 0.03 | 0.5 | 0.03 |
| 1760A0/2017 | Brazil | Rio de Janeiro | 12 | Unknown | Blood culture | 2015 | 76 | 0.25 | 0.03 | 0.5 | 0.03 |
| 295/2018 | Colombia | Medellín | 9 | Unknown | Blood culture | 2018 | 77 | 0.5 | 0.125 | 1 | 0.06 |
| 296/2018 | Colombia | Medellín | 9 | Unknown | Blood culture | 2018 | 78 | 0.25 | 0.06 | 1 | 0.06 |
| 7445/2011 | Brazil | São Paulo | 3 | Surgery | Blood culture | 2011 | 79 | 0.125 | 0.03 | 0.5 | 0.03 |
| 292/2018 | Colombia | Medellín | 9 | Unknown | Blood culture | 2018 | 80 | 1 | 0.03 | 1 | 0.03 |
| 7120/2010 | Brazil | São Paulo | 11 | Surgical ICU | Blood culture | 2010 | 81 | 0.25 | 0.03 | 1 | 0.03 |
| 7806/2011 | Brazil | São Paulo | 11 | Surgical ICU | Blood culture | 2011 | 81 | 0.25 | 0.03 | 0.5 | 0.03 |
| 1742/2017 | Brazil | Rio de Janeiro | 12 | Unknown | Blood culture | 2014 | 82 | 0.25 | 0.03 | 0.5 | 0.03 |
| 103/2019 | Brazil | São Paulo | 13 | Surgical ICU | Blood culture | 2017 | 83 | 0.25 | 0.03 | 0.25 | 0.03 |
| 1076/2016 | Brazil | São Paulo | 6 | Unknown | Catheter blood | 2016 | 84 | 0.5 | 0.06 | 0.25 | 0.03 |
| 105/2015 | Brazil | São Paulo | 18 | Unknown | Blood culture | 2015 | 85 | 0.125 | 0.03 | 0.5 | 0.06 |
| 1890/2021 | Brazil | São Paulo | 3 | General ICU adult | Blood culture | 2021 | 86 | 0.125 | 0.03 | 0.5 | 0.03 |
| CMRP5550 | Brazil | Curitiba | 2 | Unknown | Blood culture | 2022 | 87 | 0.125 | 0.06 | 0.25 | 0.03 |
| 1349/2016 | Brazil | São Paulo | 18 | Unknown | Blood culture | 2016 | 88 | 0.25 | 0.03 | 0.5 | 0.03 |
| 560/2019 | Brazil | São Paulo | 19 | Medical ICU | Blood culture | 2017 | 89 | 0.125 | 0.03 | 0.5 | 0.03 |
| 2145/2021 | Brazil | São Paulo | 18 | Unknown | Catheter blood | 2021 | 90 | 0.125 | 0.03 | 0.5 | 0.03 |
| 7416/2011 | Brazil | São Paulo | 11 | General ICU | Catheter blood | 2010 | 91 | 4 | 0.25 | 1 | 0.125 |
| 915/2019 | Brazil | São Paulo | 24 | General ICU | Blood culture | 2018 | 92 | 0.125 | 0.03 | 1 | 0.06 |
| 7225/2010 | Brazil | Curitiba | 2 | Unknown | Blood culture | 2010 | 93 | 4 | 0.25 | 0.5 | 0.03 |
| 293/2018 | Colombia | Medellín | 9 | Unknown | Blood culture | 2018 | 94 | 0.25 | 0.03 | 1 | 0.03 |
| 294/2018 | Colombia | Medellín | 9 | Unknown | Blood culture | 2018 | 94 | 0.125 | 0.03 | 1 | 0.06 |
| 1450/2017 | Brazil | Natal | Non applicable | Non applicable | Ponta Negra beach | 2017 | 95 | 4 | 0.25 | 1 | 0.06 |
| 1452/2017 | Brazil | Natal | Non applicable | Non applicable | Ponta Negra beach | 2017 | 96 | 4 | 0.25 | 1 | 0.06 |
| 1453/2017 | Brazil | Natal | Non applicable | Non applicable | Ponta Negra beach | 2017 | 96 | 4 | 0.25 | 1 | 0.06 |
| 1463/2017 | Brazil | Natal | Non applicable | Non applicable | Ponta Negra beach | 2017 | 96 | 4 | 0.25 | 1 | 0.06 |
| 1483/2017 | Brazil | Natal | Non applicable | Non applicable | Ponta Negra beach | 2017 | 96 | 4 | 0.5 | 0.5 | 0.06 |
| 1486/2017 | Brazil | Natal | Non applicable | Non applicable | Ponta Negra beach | 2017 | 96 | 4 | 0.25 | 0.5 | 0.06 |
| 1491/2017 | Brazil | Natal | Non applicable | Non applicable | Ponta Negra beach | 2017 | 96 | 4 | 0.25 | 1 | 0.06 |
| 1498/2017 | Brazil | Natal | Non applicable | Non applicable | Ponta Negra beach | 2017 | 96 | 2 | 0.25 | 0.5 | 0.06 |
| 1468/2017 | Brazil | Natal | Non applicable | Non applicable | Ponta Negra beach | 2017 | 97 | 4 | 0.25 | 0.5 | 0.06 |
| 1471/2017 | Brazil | Natal | Non applicable | Non applicable | Ponta Negra beach | 2017 | 97 | 4 | 0.25 | 1 | 0.06 |
| 1488/2017 | Brazil | Natal | Non applicable | Non applicable | Ponta Negra beach | 2017 | 97 | 2 | 0.25 | 1 | 0.06 |
| 365/2015 | Brazil | São Paulo | 3 | IU peritoneal dialysis | Blood culture | 2015 | 98 | 2 | 0.125 | 0.5 | 0.06 |
| 728/2019 | Brazil | Porto Alegre | 9 | Medical ICU | Blood culture | 2018 | 99 | 0.125 | 0.03 | 0.5 | 0.03 |
| 1760E0/2017 | Brazil | Rio de Janeiro | 12 | Unknown | Blood culture | 2017 | 100 | 0.25 | 0.06 | 0.5 | 0.03 |
| 1887/2021 | Brazil | São Paulo | 3 | General ICU adult | Blood culture | 2021 | 100 | 0.125 | 0.03 | 0.5 | 0.03 |
| CMRP5553 | Brazil | Curitiba | 2 | Unknown | Blood culture | 2022 | 101 | 0.25 | 0.03 | 0.25 | 0.03 |
| 158/2019 | Brazil | São Paulo | 18 | Unknown | Blood culture | 2019 | 102 | 0.125 | 0.03 | 1 | 0.06 |

| | | | | | | | | | | | |
|---|---|---|---|---|---|---|---|---|---|---|---|
| 1414A/2017 | Brazil | Cascavel | 25 | Unknown | Blood culture | 2016 | 103 | 0.125 | 0.03 | 0.5 | 0.03 |
| 1750A0/2017 | Brazil | Rio de Janeiro | 12 | Unknown | Blood culture | 2015 | 104 | 0.125 | 0.03 | 0.5 | 0.03 |
| 1750C0/2017 | Brazil | Rio de Janeiro | 12 | Unknown | Blood culture | 2015 | 104 | 0.125 | 0.03 | 0.5 | 0.06 |
| CMRP5543 | Brazil | Curitiba | 2 | Unknown | Blood culture | 2022 | 105 | 0.25 | 0.03 | 0.25 | 0.06 |
| CMRP5549 | Brazil | Curitiba | 2 | Unknown | Blood culture | 2022 | 106 | 0.125 | 0.03 | 0.5 | 0.03 |
| 1272/2016 | Brazil | São Paulo | 18 | Unknown | Blood culture | 2016 | 107 | 0.25 | 0.03 | 0.5 | 0.03 |
| 1270/2016 | Brazil | São Paulo | 18 | Unknown | Blood culture | 2016 | 107 | 0.125 | 0.03 | 0.5 | 0.03 |
| 645/2019 | Brazil | São Paulo | 18 | General ICU | Blood culture | 2017 | 107 | 0.125 | 0.03 | 0.5 | 0.03 |
| 652/2019 | Brazil | São Paulo | 18 | Surgical nursery | Blood culture | 2018 | 107 | 0.125 | 0.03 | 0.25 | 0.06 |
| 1879/2021 | Brazil | São Paulo | 3 | General ICU adult III | Blood culture | 2021 | 107 | 0.125 | 0.03 | 0.5 | 0.03 |
| 426/2019 | Brazil | São Paulo | 19 | Unknown | Blood culture | 2019 | 108 | 0.125 | 0.03 | 0.5 | 0.03 |
| 8095/2011 | Brazil | São Paulo | 24 | General ICU | Blood culture | 2011 | 109 | 4 | 0.125 | 0.5 | 0.06 |
| 9253/2013 | Brazil | São Paulo | 26 | Unknown | Blood culture | 2013 | 110 | 8 | 0.5 | 1 | 0.06 |
| 681/2019 | Brazil | São Paulo | 27 | Pediatric ICU | Blood culture | 2018 | 111 | 8 | 0.5 | 1 | 0.03 |
| 683/2019 | Brazil | São Paulo | 27 | Pediatric ICU | Blood culture | 2018 | 111 | 8 | 1 | 0.5 | 0.03 |
| 1541/2020 | Brazil | Salvador | 3 | IU semi intensive BMT | Blood culture | 2019 | 111 | 8 | 0.5 | 1 | 0.03 |
| 2116/2021 | Brazil | São Paulo | 6 | Unknown | Catheter blood | 2021 | 111 | 0.25 | 0.03 | 0.125 | 0.03 |
| 2118/2021 | Brazil | São Paulo | 6 | Unknown | Blood culture | 2021 | 111 | 0.25 | 0.03 | 0.5 | 0.03 |
| 2128/2021 | Brazil | São Paulo | 6 | Unknown | Catheter blood | 2021 | 111 | 0.125 | 0.03 | 0.5 | 0.03 |
| 822/2015 | Brazil | São Paulo | 3 | ICU first aid | Blood culture | 2015 | 112 | 8 | 1 | 0.5 | 0.06 |
| 1743/2017 | Brazil | Rio de Janeiro | 12 | Unknown | Blood culture | 2017 | 112 | 8 | 0.5 | 0.5 | 0.03 |
| 1535/2017 | Brazil | Salvador | 3 | Unknown | Blood culture | 2017 | 113 | 8 | 0.5 | 0.5 | 0.03 |
| 1034/2020 | Brazil | São Paulo | 18 | Unknown | Catheter blood | 2020 | 114 | 0.25 | 0.03 | 0.5 | 0.03 |
| 1086/2020 | Brazil | São Paulo | 18 | Unknown | Blood culture | 2020 | 114 | 0.25 | 0.03 | 0.125 | 0.03 |
| 1540/2020 | Brazil | Salvador | 3 | IU semi intensive BMT | Blood culture | 2019 | 115 | 8 | 0.5 | 1 | 0.03 |
| 1534/2017 | Brazil | Salvador | 3 | Unknown | Blood culture | 2017 | 116 | 8 | 0.5 | 0.5 | 0.03 |
| 7553/2011 | Brazil | Curitiba | 28 | Medical clinic | Catheter blood | 2010 | 117 | 0.25 | 0.03 | 00.5 | 0.03 |
| 7564/2011 | Brazil | São Paulo | 11 | Orthopedics | Blood culture | 2011 | 117 | 0.25 | 0.03 | 0.5 | 0.03 |
| 1833/2021 | Brazil | São Paulo | 3 | IU gastroclinic | Blood culture | 2020 | 118 | 0.125 | 0.03 | 0.25 | 0.03 |
| 1834/2021 | Brazil | São Paulo | 3 | IU gastroclinic | Catheter blood | 2020 | 118 | 0.125 | 0.03 | 0.5 | 0.03 |
| 6926/2010 | Brazil | Salvador | 3 | Pediatric ICU | Blood culture | 2010 | 119 | 0.125 | 0.03 | 0.5 | 0.03 |
| 251/2019 | Brazil | São Paulo | 19 | Unknown | Blood culture | 2018 | 120 | 0.25 | 0.03 | 0.5 | 0.03 |
| 401/2015 | Brazil | São Paulo | 3 | ICU adult first aid | Blood culture | 2015 | 121 | 0.25 | 0.03 | 0.5 | 0.03 |
| 10101/2014 | Brazil | São Paulo | 3 | Observation unit adult first aid | Catheter blood | 2014 | 122 | 0.5 | 0.03 | 0.5 | 0.03 |
| 10102/2014 | Brazil | São Paulo | 3 | Observation unit adult first aid | Blood culture | 2014 | 122 | 0.5 | 0.03 | 0.5 | 0.03 |
| 1910/2017 | Brazil | São Paulo | 18 | Unknown | Blood culture | 2017 | 123 | 0.125 | 0.03 | 0.5 | 0.03 |
| 10177/2014 | Brazil | São Paulo | 3 | ICU pediatrics | Blood culture | 2014 | 124 | 0.125 | 0.03 | 0.5 | 0.03 |
| 10/2015 | Brazil | São Paulo | 3 | ICU pediatrics | Blood culture | 2014 | 124 | 0.25 | 0.06 | 0.5 | 0.03 |
| CMRP5545 | Brazil | Curitiba | 2 | Unknown | Blood culture | 2022 | 125 | 0.5 | 0.03 | 0.125 | 0.06 |
| 1243/2020 | Brazil | São Paulo | 3 | Observation unit adult first aid | Blood culture | 2020 | 126 | 0.25 | 0.03 | 0.5 | 0.06 |
| 7171/2010 | LATAM | Unknown | 29 | Unknown | Blood culture | 2010 | 127 | 0.25 | 0.03 | 1 | 0.06 |
| 613/2019 | Brazil | São Paulo | 19 | Unknown | Blood culture | 2018 | 128 | 0.25 | 0.03 | 0.5 | 0.03 |
| 498/2019 | Brazil | São Paulo | 13 | Clinical nursery | Blood culture | 2017 | 129 | 0.25 | 0.03 | 0.25 | 0.03 |
| 555/2019 | Brazil | São Paulo | 19 | General ICU | Blood culture | 2018 | 130 | 0.25 | 0.03 | 0.5 | 0.03 |
| 583/2019 | Brazil | São Paulo | 19 | General ICU | Blood culture | 2018 | 130 | 0.25 | 0.03 | 0.5 | 0.03 |
| 584/2019 | Brazil | São Paulo | 19 | General ICU | Blood culture | 2018 | 130 | 0.25 | 0.03 | 0.25 | 0.03 |
| 6876A/2010 | Brazil | Rio de Janeiro | 15 | Unknown | Blood culture | 2010 | 131 | 0.125 | 0.03 | 0.5 | 0.03 |
| 750/2019 | Brazil | Porto Alegre | 4 | General ICU | Blood culture | 2018 | 132 | 0.125 | 0.03 | 0.5 | 0.03 |
| 751/2019 | Brazil | Porto Alegre | 4 | General ICU | Blood culture | 2018 | 132 | 0.125 | 0.03 | 0.5 | 0.03 |
| 155/2018 | Brazil | São Paulo | 19 | ICU emergency room adult | Catheter blood | 2018 | 133 | 0.125 | 0.03 | 0.5 | 0.03 |
| CMRP5541 | Brazil | Curitiba | 2 | Unknown | Blood culture | 2022 | 134 | 0.25 | 0.03 | 0.125 | 0.06 |
| 6876B/2010 | Brazil | Rio de Janeiro | 15 | Unknown | Blood culture | 2010 | 135 | 0.125 | 0.03 | 0.5 | 0.03 |
| 216/2019 | Brazil | São Paulo | 19 | General ICU | Blood culture | 2018 | 136 | 0.125 | 0.03 | 0.5 | 0.03 |

| | | | | | | | | | | | |
|---|---|---|---|---|---|---|---|---|---|---|---|
| 612/2019 | Brazil | São Paulo | 19 | General ICU | Blood culture | 2018 | 137 | 0.25 | 0.03 | 0.25 | 0.03 |
| 701/2019 | Brazil | São Paulo | 27 | Unknown | Blood culture | 2018 | 138 | 0.125 | 0.03 | 0.5 | 0.03 |
| 703/2019 | Brazil | São Paulo | 27 | Unknown | Blood culture | 2018 | 139 | 0.25 | 0.03 | 0.25 | 0.03 |
| 8094/2011 | Brazil | São Paulo | 24 | ICU | Blood culture | 2011 | 140 | 0.25 | 0.03 | 0.5 | 0.03 |
| 685/2019 | Brazil | São Paulo | 27 | Surgical nursery | Catheter blood | 2017 | 141 | 0.125 | 0.03 | 0.25 | 0.03 |
| 102/2019 | Brazil | Rio de Janeiro | 15 | Unknown | Blood culture | 2017 | 142 | 0.125 | 0.03 | 0.5 | 0.03 |
| 730/2019 | Brazil | Porto Alegre | 4 | General ICU | Catheter blood | 2018 | 143 | 0.125 | 0.03 | 0.5 | 0.03 |
| 99/2019 | Brazil | São Paulo | 3 | General ICU | Blood culture | 2017 | 144 | 0.125 | 0.03 | 0.5 | 0.03 |
| 101/2019 | Brazil | São Paulo | 19 | General ICU | Blood culture | 2017 | 144 | 0.125 | 0.03 | 0.5 | 0.03 |
| 1556/2017 | Brazil | Londrina | 1 | Unknown | Blood culture | 2017 | 145 | 0.125 | 0.03 | 0.25 | 0.03 |
| 1734D0/2017 | Brazil | Rio de Janeiro | 12 | Unknown | Blood culture | 2011 | 146 | 0.125 | 0.03 | 0.5 | 0.03 |
| 1735B0/2017 | Brazil | Rio de Janeiro | 12 | Unknown | Blood culture | 2011 | 146 | 0.125 | 0.03 | 0.5 | 0.03 |
| 6317/2010 | Brazil | São Paulo | 19 | Unknown | Blood culture | 2010 | 147 | 0.25 | 0.03 | 1 | 0.03 |
| 6395/2010 | Brazil | São Paulo | 19 | Unknown | Blood culture | 2010 | 147 | 0.25 | 0.03 | 0.5 | 0.03 |
| 985/2019 | Brazil | São Paulo | 18 | Unknown | Catheter blood | 2019 | 148 | 0.125 | 0.03 | 0.5 | 0.03 |
| 1019/2019 | Brazil | São Paulo | 3 | Medical urgency | Blood culture | 2019 | 149 | 0.5 | 0.03 | 0.5 | 0.03 |
| 1023/2019 | Brazil | São Paulo | 3 | Medical urgency | Blood culture | 2019 | 149 | 0.5 | 0.03 | 0.5 | 0.03 |
| 6966/2010 | Brazil | São Paulo | 3 | Burn unit | Blood culture | 2010 | 150 | 0.125 | 0.03 | 1 | 0.03 |
| 8030/2011 | Brazil | Porto Alegre | 4 | Emergency | Blood culture | 2011 | 151 | 0.25 | 0.03 | 0.25 | 0.03 |
| 7393A/2011 | Brazil | Belo Horizonte | 5 | Surgery | Blood culture | 2011 | 152 | 0.125 | 0.03 | 0.5 | 0.03 |
| 1131/2016 | Brazil | Londrina | 7 | Unknown | Catheter blood | 2016 | 153 | 0.5 | 0.06 | 0.5 | 0.03 |
| 1148/2016 | Brazil | Londrina | 1 | Unknown | Blood culture | 2016 | 154 | 0.125 | 0.03 | 0.25 | 0.03 |
| 1746/2017 | Brazil | Rio de Janeiro | 12 | Unknown | Blood culture | 2015 | 155 | 0.25 | 0.03 | 0.5 | 0.03 |
| 1152/2016 | Brazil | Londrina | 1 | Unknown | Blood culture | 2016 | 156 | 0.125 | 0.03 | 0.5 | 0.03 |
| 1859/2017 | Brazil | Maringá | 30 | Unknown | Blood culture | 2017 | 157 | 0.25 | 0.03 | 0.5 | 0.03 |
| 1100/2016 | Brazil | São Paulo | 19 | ICU emergency room adult | Catheter blood | 2016 | 158 | 1 | 0.125 | 0.5 | 0.03 |
| 1751/2017 | Brazil | Rio de Janeiro | 12 | Unknown | Blood culture | 2015 | 159 | 0.5 | 0.06 | 1 | 1 |
| 303/2015 | Brazil | São Paulo | 6 | Unknown | Blood culture | 2015 | 160 | 0.25 | 0.03 | 0.5 | 0.03 |
| 566/2015 | Brazil | São Paulo | 3 | IU cardiology | Blood culture | 2015 | 161 | 0.25 | 0.03 | 0.5 | 0.03 |
| 987/2019 | Brazil | São Paulo | 6 | Unknown | Blood culture | 2019 | 162 | 0.5 | 0.03 | 0.5 | 0.03 |
| 989/2019 | Brazil | São Paulo | 6 | Unknown | Blood culture | 2019 | 162 | 0.5 | 0.03 | 0.5 | 0.03 |
| CMRP5547 | Brazil | Curitiba | 2 | Unknown | Blood culture | 2022 | 163 | 0.125 | 0.03 | 0.25 | 0.03 |
| 1423/2020 | Brazil | São Paulo | 18 | Unknown | Catheter blood | 2020 | 164 | 0.125 | 0.03 | 0.5 | 0.03 |
| 1424/2020 | Brazil | São Paulo | 18 | Unknown | Blood culture | 2020 | 164 | 0.125 | 0.03 | 0.5 | 0.03 |

* Catheter blood is a blood culture collected via a central venous catheter. Blood culture is blood obtained via a vena puncture.
