# Supplementary figures and images for: Short Tandem Repeat Genotyping and Antifungal Susceptibility Testing of Latin American Candida tropicalis Isolates
Source: J Fungi (Basel). 2023 Feb 5;9(2):207. doi: 10.3390/jof9020207 (PMC9958743; doi:10.3390/jof9020207)

## Slide 1
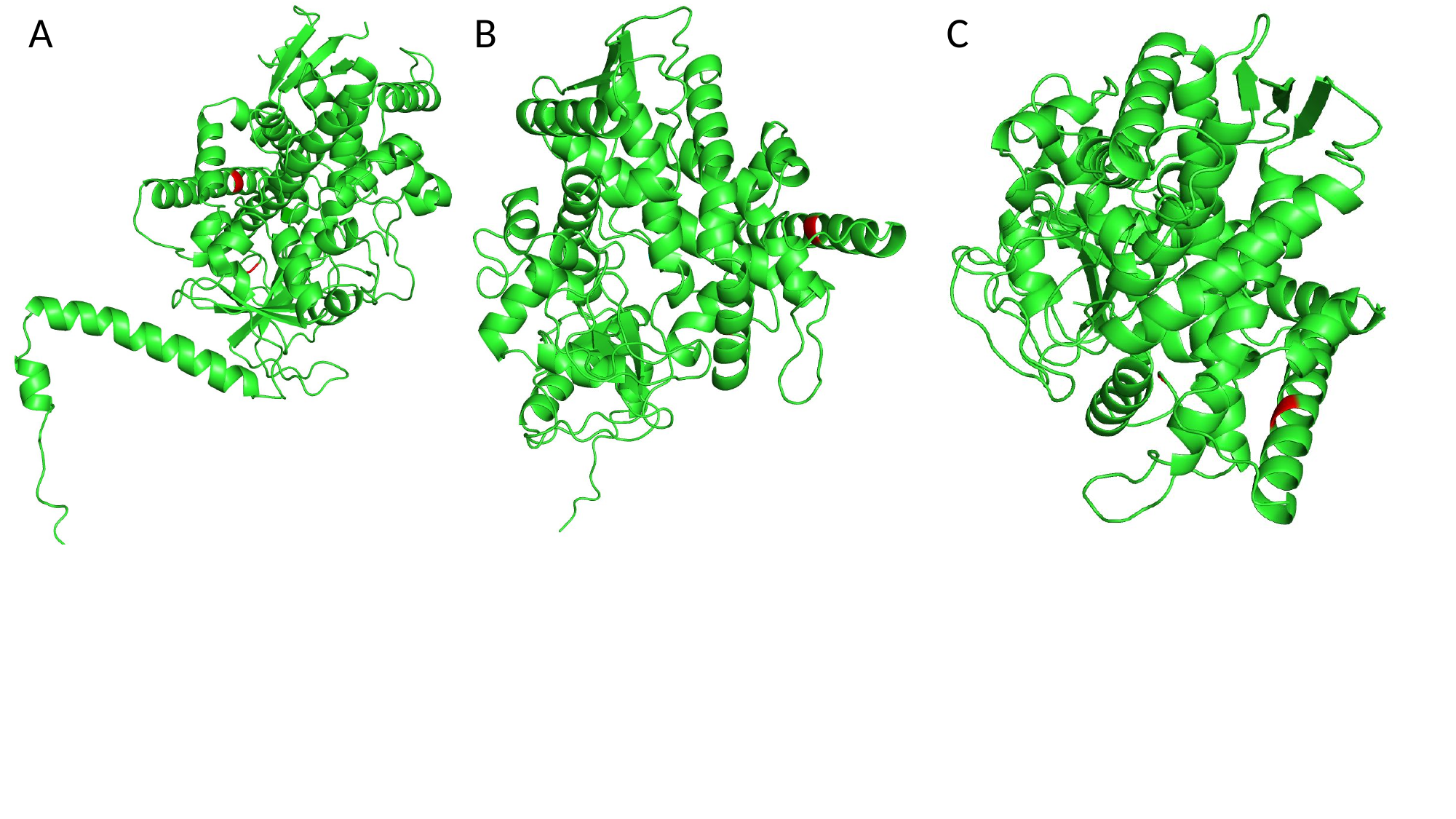

A
B
C

Supplement: Supplementary file 1 [file jof-09-00207-s001.zip › jof-2189999-supplementary/Supplementary Figure S2 STR Cantro Brazil.pptx]
